# Heterogeneity of triple negative breast cancer: Current advances in subtyping and treatment implications

**DOI:** 10.1186/s13046-022-02476-1

**Published:** 2022-09-01

**Authors:** Karama Asleh, Nazia Riaz, Torsten O. Nielsen

**Affiliations:** 1 Genetic Pathology Evaluation Centre, Department of Pathology and Laboratory Medicine, University of British Columbia, Vancouver, Canada; 2 Interdisciplinary Oncology Program, Faculty of Medicine, University of British Columbia, Vancouver, Canada; 3 Department of Pathology and Laboratory Medicine, Vancouver General Hospital, 899 West 12th Avenue, Vancouver, BC V5Z 1M9 Canada

**Keywords:** Tripe negative breast cancer, Molecular subtyping, Genomics, Proteomics, Single cell profiling, Biomarkers, Clinical trials, Therapeutic targets

## Abstract

As the field of translational ‘omics has progressed, refined classifiers at both genomic and proteomic levels have emerged to decipher the heterogeneity of breast cancer in a clinically-applicable way. The integration of ‘omics knowledge at the DNA, RNA and protein levels is further expanding biologic understanding of breast cancer and opportunities for customized treatment, a particularly pressing need in clinically triple negative tumors. For this group of aggressive breast cancers, work from multiple groups has now validated at least four major biologically and clinically distinct omics-based subtypes. While to date most clinical trial designs have considered triple negative breast cancers as a single group, with an expanding arsenal of targeted therapies applicable to distinct biological pathways, survival benefits may be best realized by designing and analyzing clinical trials in the context of major molecular subtypes. While RNA-based classifiers are the most developed, proteomic classifiers proposed for triple negative breast cancer based on new technologies have the potential to more directly identify the most clinically-relevant biomarkers and therapeutic targets. Phospho-proteomic data further identify targetable signalling pathways in a unique subtype-specific manner. Single cell profiling of the tumor microenvironment represents a promising way to allow a better characterization of the heterogeneity of triple negative breast cancer which could be integrated in a spatially resolved context to build an ecosystem-based patient classification. Multi-omic data further allows in silico analysis of genetic and pharmacologic screens to map therapeutic vulnerabilities in a subtype-specific context. This review describes current knowledge about molecular subtyping of triple negative breast cancer, recent advances in omics-based genomics and proteomics diagnostics addressing the diversity of this disease, key advances made through single cell analysis approaches, and developments in treatments including targeted therapeutics being tested in major clinical trials.

## Introduction

Over the past 20 years, breast cancer has been recognized to be a family of diseases with distinct pathological, molecular, and clinical features. Several classification systems have been proposed to represent the biology underpinning different manifestations of breast cancer that influence prognosis and treatment. Because effective, well-established targeted therapies exist for estrogen/progesterone receptor positive (luminal) and Her2 receptor positive subtypes, research efforts are being focused on gaining a better understanding of the biology of triple negative breast cancers (TNBC). These account for approximately 15% of cases, afflict younger women [1], are clinically aggressive and associated with early recurrences [2, 3].

TNBC is a clinical definition of convenience based on negative staining for three standard immunohistochemical (IHC) biomarkers, and their associated lack of obvious systemic targeted (endocrine or anti-Her2) treatments. Thus, it is not surprising that TNBC delineates a group with heterogeneous biology [4–7]. Indeed, the field of translational ‘omics has seen advances over the past decade that recognize at least four biologically and clinically-distinct TNBC subtypes. Many challenges impede the facile conversion of omics-based classifiers into assays with clinical utility [8], and TNBC subtyping tests have yet to become a routine part of clinical care. Cytotoxic chemotherapy remains the mainstay of systemic treatment for TNBC [9], supplemented by a still-limited set of targeted therapeutics that can improve overall survival (OS) for metastatic TNBC patients beyond a median of 15 months [10–12]. This is partly attributable to current clinical trial designs that indiscriminately lump together all TNBCs, ignoring subtypes that have critical biological and immunological differences, but done to hasten accrual and obtain results applicable to a larger target market should the trial read out as positive.

Nonetheless, lessons learned over the past two years including well-designed exploratory analyses of two major immunotherapy trials [13, 14] have highlighted the need to account for distinct molecular TNBC subtypes and find better ways to match TNBC patients to their best treatment.

In this review, we detail recent advancements in genome-wide DNA, RNA, and protein-based classifications of TNBC, discuss their utility in translational oncology in the context of recent developments from major clinical trials assessing chemotherapy and targeted therapy, and highlight the importance of integrating the tumor microenvironment in current TNBC subtyping to uncover relational features of tumor, stromal and immune cells as revealed by recent single cell profiling studies.

### DNA-level genomic insights into triple negative breast cancer

Building on the cDNA microarray-based work first described by Perou et al.[15], the existence of luminal A, luminal B, Her2-Enriched and basal-like breast cancer intrinsic subtypes has been recapitulated using different gene expression platforms and independent datasets [16–22]. Of these, the basal-like gene expression subtype is distinctly different from others in biology and clinical outcomes [15, 23–26]. In current clinical practice, this subtype is still approximated by TNBC IHC status, which identifies a heterogeneous biology [4–7]. Between 50–86% of TNBC have been shown to be basal-like by gene expression [11, 17, 26–28], meaning the terms should not be considered interchangeable.

While the genomic landscape of breast cancer in large-scale studies including The Cancer Genome Atlas (TCGA) [26] and Molecular Taxonomy of Breast Cancer International Consortium (METABRIC) [28] has been mainly characterized in the context of breast cancer gene expression subtypes, the genomic landscape of TNBC has been specifically investigated in studies using whole genome, whole exome or targeted sequencing [25, 29–33]. Several somatic mutation patterns have been described in TNBC, which has the highest overall mutation rate when compared to other forms of breast cancer [26]. This higher mutational burden is nevertheless characterized by very few consistently mutated genes other than *TP53*, highlighting the complex repertoire of somatic mutations that underpin the heterogeneity of TNBC [25, 26, 34]. Thus, developing clinically-useful biomarkers targeting the numerous low-frequency genetic events of TNBC is a considerable challenge. TNBC as a group is characterized by a complex pattern of copy number alterations implying higher chromosomal instability when compared to other subtypes [28, 35]. These patterns have been described as a sawtooth, with many small and narrow segments of deletions and duplications, but not involving high copy number amplifications [36]. *EGFR*, *FGFR2* and *MYC* amplifications as well as *PTEN* losses are more frequent in TNBC [25, 29, 35] but appear to be more clinically-relevant when analyzed in the context of TNBC transcriptomic subtypes [6, 35].

Mutational analyses of the somatic alteration repertoire of breast cancer have contributed to the understanding of the mutational processes that characterize TNBC [31, 32, 37]. Catalogue of Somatic Mutations in Cancer (COSMIC) substitution-based mutational signatures [38, 39] of homologous recombination DNA repair deficiency (HRD) signatures (COSMIC 3 and 8) are known to be more characteristic of TNBC when compared to clock-like (1 and 5) and APOBEC signatures that dominate among luminal breast cancers [31, 32, 37]. The mutational processes underlying the high genomic instability and HRD in TNBC can be further captured as patterns of indels and structural variants known as rearrangement signatures (RS) [31]. Of these RS-1, RS-3 and RS-5 have been associated with HRD in TNBC with the advantage of subclassifying tumors according to their *BRCA1*/*2* mutation status [31]. Small tandem duplications (< 10 kb, RS-3) are associated with *BRCA1*-mutation vs. large tandem duplications (> 100 kb, RS-1) which are more characteristic of *BRCA* wild-type *TP53*-mutated [31–33]. Sizes of tandem duplications further define biological classes as part of a distinct genomic scar enriched in TNBC known as tandem duplicator phenotype (TDP) [40]. The TDP-1 biological class (< 10 kb) is associated with *BRCA1* mutated TNBC, but not *BRCA2* [40]. High TDP scores have been linked with response to platinum-based chemotherapy in TNBC preclinical models; thus, TDP may serve as a predictive genomic biomarker [40].

Several tests have been developed to characterize HRD in TNBC given that *BRCA1* germline mutations are found in approximately 15% of these tumors and most *BRCA1*-mutated tumors are of the TNBC subtype [41–47]. TNBC is known to display several BRCAness features through somatic *BRCA1/2* mutations or germline/somatic alterations in homologous repair-related genes (e.g., *PALB2*, *ATM*, *CHEK2*) [48, 49], characterized by high scores using the copy number based HRD algorithm [50, 51] or the genomic-signature based HRDetect [32, 52, 53]. Epigenetic silencing of *BRCA1* and *RAD51C* is also associated with BRCAness [54] with *BRCA1* promoter methylation being exclusive to TNBC [55]. The HRDetect algorithm covers several patterns of BRCAness combining features of HRD index (loss of heterozygosity, telomeric allelic imbalance, and large-scale transitions); COSMIC substitution signatures 3 and 8; RS-3 and RS-5 signatures; and microhomology-mediated deletions to classify TNBC into HRDetect-high, HRDetect-intermediate, and HRDetect-low with the HRDetect-high subgroup associated with a better response to standard chemotherapy [32, 52]. When compared to HRD index, HRDetect has demonstrated superior performance to detect *BRCA1/2* deficiency [52], a rationale that informed the evaluation of HRDectect as a predictive biomarker for carboplatin response in the phase III TNT trial of advanced TNBC, where results using the HRD index scores were negative [56].

Overall, the complexity of TNBC at both the copy number and mutational levels with many and variable low-frequency genomic events that contribute to diverse sub-clonal populations explains in part the lack of current genomic-based clinical tests to guide therapeutic choices. However, the integration of these data, especially HRD-based signatures in the context of transcriptomic TNBC subtypes, could aid in developing genomic aberration-based biomarkers in a TNBC subtype-specific manner.

### Transcriptomic heterogeneity of triple negative breast cancer

Applying RNA expression profiling to TNBC cases, several molecular systems have been suggested to identify subtypes and associated biomarkers that guide treatment decisions.

In 2011, Lehmann and colleagues performed gene expression profiling of 587 TNBCs and postulated the existence of six main transcriptional subtypes: basal-like 1, basal-like 2, immunomodulatory, luminal androgen receptor (LAR), mesenchymal and mesenchymal stem-like (MSL) [4]. A follow-up study in 2016 refined these into four subtypes [57] because after a careful histological evaluation and microdissection prior to gene expression analysis, the transcriptional profiles of immunomodulatory and MSL were found to reflect tumor infiltrating lymphocytes and stromal content, rather gene expression by carcinoma cells. The revised Lehmann TNBC-type4 classification includes basal-like 1, basal-like 2, mesenchymal and LAR categories, among which basal-like 1 had a higher pathologic complete response rate (pCR) after neoadjuvant chemotherapy and a better OS [57, 58]. However, a study by Bareche and colleagues [35] which analyzed 550 TNBC samples from both TCGA and METABRIC using the original Lehmann molecular subtyping [4] still described five molecular subtypes. After exclusion and reclassification of “unstable” samples through bioinformatics methods applied to the online TNBCtype tool used in Lehmann’s original study, the authors reported that TNBC can be classified as basal-like 1, immunomodulatory, mesenchymal, LAR, and MSL [35]. However, this revised 5-subtype classification requires assessment and validation on independent datasets.

Another TNBC molecular classification was first presented by Burstein and colleagues in 2015 who performed mRNA expression and DNA profiling on 198 TNBC tumors, identifying four transcriptomic subtypes termed basal-like immune activated (BLIA), basal-like immune suppressed (BLIS), LAR, and mesenchymal [7]. These subtypes were identified using a reduced 80-gene RNA-based classifier, were shown to differ in survival and to exhibit distinctive patterns of copy number variations, gene expression and pathway enrichment [7]. Among these four categories, survival outcomes were most favorable for BLIA and worst for BLIS.

The 4 TNBC subtypes in Burstein et al.were recapitulated on an independent larger cohort of 504 Chinese TNBC cases [6]. Compared to the TCGA dataset, this study highlighted distinct patterns in the Chinese TNBC population including higher frequencies of *PIK3CA* mutations and LAR subtype. Zhao et al.subsequently utilized the RNA-sequencing data from this study to develop an IHC-based classifier on AR, CD8, FOXC1 and DCLK1 [59], although this lacks external independent validation yet.

**Fig. 1:**
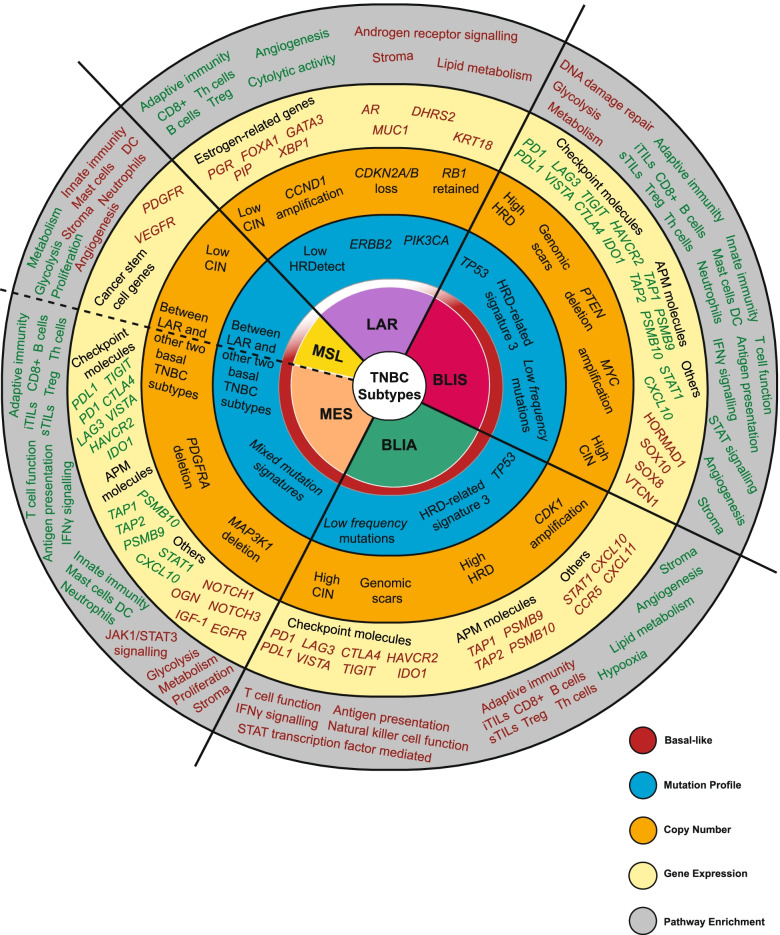
Overview of the characteristic mutation profile, copy number, gene expression and pathway enrichment for the different triple negative breast cancer subtypes Triple negative breast cancer subtypes of luminal androgen receptor (LAR), basal-like immune suppressed (BLIS), basal-like immune activated (BLIA), mesenchymal (MES) and the possible addition of mesenchymal stem-like (MSL) are shown at the core. Genes and pathways denoted in red (indicate high expression) while those denoted in green (indicate low expression). Data are aggregated from Lehmann et al.[57], Burstein et al.[7], Jiang et al.[6], Bareche et al.[35], Bareche et al.[64], Lehmann et al.[58], Gong et al.[76] and Asleh et al.[60]. Abbreviations: TNBC, triple negative breast cancer; HRD,

Altogether, based on independent DNA and RNA level analyses, the current consensus is that TNBC comprises of at least four major molecular subtypes categorized as BLIA (20–30%), BLIS (25–40%), LAR (15–25%), and mesenchymal (15–20%) [6, 7, 35]. Characteristic mutation profile, copy number, gene expression and pathway enrichment of the different TNBC subtypes are depicted in Fig. 1.

#### BLIA: Basal Like Immune Activated Subtype

The mutational profile of BLIA demonstrates that > 80% of these tumors harbor *TP53* mutations, with otherwise a high number of individually low-frequency mutations [6, 35]. BLIA is characterized by high chromosomal instability scores and enrichment for DNA damage repair processes [6, 7, 35]. *CDK1* amplification is more characteristic of this subtype than others [7]. 

BLIA cancers highly express genes involved in immune-response pathways including T cell function, antigen processing and presentation, interferon (IFN)γ signalling [6, 7, 35, 60] and genes encoding checkpoint molecules such as *CTLA4*, *PD1*, and *PDL1* [6, 7, 35, 60]. On hematoxylin and eosin histologic sections, intratumoral and stromal lymphocytes are significantly higher in BLIA compared to other TNBC subtypes [6, 60]. Several recently-developed RNA-based signatures in the TONIC [61], KEYNOTE-086 [62] and BRAVO-Dx IMMUNE [63] trials support the existence of an immune microenvironment that makes BLIA the subtype most appropriate for immune checkpoint inhibitors. BLIA tumors have been further reported to be depleted for stromal, angiogenesis and metabolic processes [60, 64] linked to immunotherapy resistance [65–67].

#### BLIS: Basal Like Immune Suppressed Subtype

BLIS is the most common TNBC subtype [6, 7, 35] and, similar to BLIA, displays high chromosomal instability scores, enrichment for DNA damage repair pathways, *TP53* loss and a high total number of low-frequency mutations overall [6, 7, 35]. However, BLIS is characterized by minimal expression of immune-response pathways, suggesting that these tumors can be immunologically ignorant or excluded [7, 60], making them unlikely to respond to immunotherapy [61]. BLIS represents a subset of breast cancers linked with high recurrence rates [63] and has been shown to highly express *VTCN1* which encodes for B7-H4 that negatively regulates T cell activation [6, 7, 60, 68]. Furthermore, BLIS tumors express multiple transcription factors of the *SOX* family that promote tumor proliferation and invasion [7]. In contrast to BLIA tumors, BLIS are enriched for pathways involved in metabolic reprogramming [60, 64] that confer cell proliferation [69], and resistance for immunotherapy [65, 70]**.**

BLIS tumors have been characterized as enriched for HRD signatures and genomic scars when compared to other TNBC subtypes, with those classified as high-HRD having a better prognosis when compared to low-HRD [6]**.** RAD51-low score during S/G2 cell cycle phase has shown 87% concordance with functional HRD status and predicts pCR following platinum-based neoadjuvant chemotherapy in unselected TNBC [71]. Increases in RAD51 nuclear foci between baseline and end of treatment biopsies further indicate resistance to neoadjuvant poly (ADP-ribose) polymerase (PARP) inhibitors [72]. Whether RAD51-low score is a useful biomarker for BLIS tumors needs further investigation. 

#### LAR: Luminal Androgen Receptor Subtype

While LAR breast cancers are negative for ER expression by IHC, they are still characterized by high expression of estrogen-related genes (e.g., *FOXA1*, *GATA3*, *PGR* and *XBP1*), *PIP*, *MUC1* and increased androgen receptor signalling [6, 7, 57, 58]. Most of these tumors are profiled as non-basals (luminal and Her2-Enriched) by PAM50 [6, 7, 57, 60]. While anti-tumor activity has been demonstrated by targeting androgen signalling in preclinical TNBC models [58], completed phase I/II trials have to date shown limited efficacy with agents targeting androgens in TNBC [73]. While the LAR subtype is clinically characterized as negative for Her2 by IHC expression and fluorescence in situ hybridization, it carries more frequent *ERBB2* mutations when compared to other TNBC subtypes [6, 58, 74]. These are mainly activating mutations in the *ERBB2* kinase domain, known to induce a relative activation of the *ERBB2* pathway but associated with resistance to trastuzumab [6], a finding that supports using alternate treatments for LAR such as tyrosine kinase inhibitors [27]. LAR tumors further display a somatic mutational pattern frequently found in luminal tumors, mainly *PIK3CA* mutations in approximately 40–55% [6, 35]. The hyperactivity of PI3K/AKT pathway in LAR is further demonstrated by increased phosphorylation of AKT1 and AKT2 [58].

Preclinical models have shown that LAR benefits from PI3K-AKT inhibitors and their combination with CDK4/6 inhibitors, with a greater efficacy observed among *PIK3CA*-mutant tumors when compared to wild-type [74]**.** Additionally, LAR tumors are characterized by *CDKN2A* loss while retaining *RB1* and have high phosphorylation of RB protein supporting their sensitivity to CDK4/6 inhibitors [58, 74, 75]. LAR tumors further exhibit low levels of chromosomal instability, HRD and immune-related gene expression [6, 7, 32, 33, 35, 58] and have been associated with lipid metabolism [58, 60, 64, 76], older age, positive lymph nodes, and apocrine features [6, 57, 58].

#### Mesenchymal Subtype

The mesenchymal subtype of TNBC is enriched for pathways involved in extracellular matrix, epithelial-mesenchymal-transition and angiogenesis [6, 7, 58, 60, 76] expressing *EGFR*, *IGF-1*, osteocyte markers (*OGN*), *NOTCH1* and *NOTCH3* [6, 35, 58]. Mesenchymal tumors as a group display mixed basal-like and non-basal PAM50 profiles [6]. Their mutational profile has proven to be hard to characterize and is reported to be intermediate between LAR and the two basal TNBC subtypes, with a mix of mutation signatures [6]. Recently, mesenchymal tumors have been reported to display higher genomic instability, tumor mutation burden and copy number alterations than other subtypes, while characterized by low immune cells and PDL1 expression, suggesting that they have developed immune-evasive mechanisms contributing to their resistance to immunotherapy [58]. 

Higher fractions of copy number deletions in *MAP3K1*, *PDGFRA*, genes involved in DNA repair and antigen presentation and processing (*B2M*) have been reported among mesenchymal tumors when compared to other TNBCs [35, 58]. Epigenetic modifier mutations that regulate the polycomb repressive complex 2 (PRC2) such as those in the *ASXL* gene family are enriched in mesenchymal tumors [58]. Loss of function mutations in the BAF SWI/SNF complex that increases the activity of PRC2 further leads to transcriptional repression of antigen presentation and processing genes, potentially rendering mesenchymal tumors sensitive to EZH2 inhibitors [58, 77]. When compared to other TNBC subtypes, mesenchymal tumors display DNA hypermethylation and concordant low expression of immune-related genes while being characterized with the most hypomethylated CpGs dispersed throughout the chromosomes [58]. EZH2 inhibitors demethylate immune-response and antigen presentation promoter regions, restoring major histocompatibility complex-I expression which could be used as a strategy to sensitize mesenchymal tumors to chemotherapy and immunotherapy [58].

While antigen processing and presentation proteins are low in mesenchymal tumors [58, 60, 76], phospho-proteomic analysis has shown an increased DNA repair signalling in this subtype as demonstrated by high phosphorylation of ATR and ATM [58]. Mesenchymal tumors have been further reported to display characteristics of breast cancer stem cells and upregulation in JAK1/STAT3 signalling [35]. While JAK1 inhibitors have shown negative results among unselected metastatic TNBC patients [78], they might have value specifically in mesenchymal tumors. An epithelial-mesenchymal-transition signature-enriched profile has recently been reported to predominate in TNBC not achieving pCR [79].

When compared to mesenchymal, at least half of MSL tumors consist of non-basal PAM50 profiles and are more enriched for angiogenesis signatures [35]. MSL further displays the highest expression of *VEGF* and *PDGFR* when compared to other subtypes, implying that MSL might benefit the most from anti-angiogenic therapies. While anti-angiogenic drugs have shown negative results in unselected TNBC [80], they might be a good option for MSL which is further enriched for innate immune cells [64] (e.g. mast cells) that induce tumor angiogenesis [81–83].

### Heterogeneity of triple negative breast cancer as revealed by proteomics

Despite advances in genomic classifications, extensive heterogeneity still characterizes TNBCs beyond their DNA and RNA profiles, with most clinical tests and treatment decisions based on protein level information. New proteomic classifications have been used to investigate the functional differences underpinning breast cancer heterogeneity and develop even more clinically-relevant classifiers. For breast cancer in general, two proteomic classifications using fresh frozen materials have been recently proposed [84–86].

The first is the mass spectrometry proteomic characterization of breast cancer by the Clinical Proteomic Tumor Analysis Consortium (CPTAC) originally published in 2016 [84]. This study profiled 77 TCGA breast cancer specimens, demonstrating three distinct proteome subgroups. Two of them recapitulated the luminal and basal PAM50 RNA subtypes, while a third subgroup termed as “stromal-enriched” consisted of a mix of PAM50 subtypes. Incorporating comprehensive characterization of the proteome, phospho-proteome and acetylproteome, this classification was refined in 2020 by CPTAC, analyzing fresh frozen materials from 122 TCGA breast cancer specimens and describing the presence of four proteome subgroups which correlated with their PAM50 subtypes, termed “LumA-Inclusive”, “LumB-Inclusive”, “Basal-Inclusive”, “Her2-Inclusive” [84]. Acetylproteome profiling revealed basal-specific metabolic pathways characterized by hyperacetylation of proteins involved in the TCA cycle and beta-oxidation enzymes that could serve as therapeutic vulnerabilities [84]. While the proteomic analysis in this study did not distinguish subtypes within TNBC as a group, phospho-proteomic analysis revealed two distinct subsets within 28 TNBC cases based on retinoblastoma protein (RB) phosphorylation status [84]. TNBC with *RB1* mutations/deletions had lower levels of RB phosphorylation when compared to wild-type, proposing that integrating proteogenomics could better define biomarkers for response to CDK4/6 inhibitors [84].

The second study is a mass spectrometry-based proteomic classification published in 2019 that analyzed nine fresh frozen breast cancer specimens from each of the five major PAM50 subtypes, using the OSLO2 cohort [86]. This study revealed a further heterogeneity in breast cancers when compared to CPTAC, describing six proteome subtypes termed consensus core tumor clusters. Overall, these consensus clusters highly correlated with their corresponding PAM50 RNA categorizations as luminal A, normal-like and basal-like while partially recapitulating the Her2-Enriched and Luminal B PAM50 subtypes. The OSLO2 proteomic classification defined two distinct clusters within basal-like as “basal immune hot” (*n* = 2) vs. “basal immune cold” (*n* = 7) [86].

Very recent attempts aimed to specifically define the proteomic heterogeneity of TNBC. A mass spectrometry-based analysis of 90 fresh frozen TNBC specimens based on global proteomics and phospho-proteomics integrated with genomic data revealed four main biologically and clinically-distinct subgroups that had key pathway and protein expression characteristics similar to the established transcriptomic BLIA, BLIS, LAR, and mesenchymal subtypes profiled on the same cohort [76]. This classifier further revealed potential therapeutic targets specifically for the proteome subgroup that resembled LAR; these were involved in fatty acid metabolism (e.g. FASN), recently proposed as a promising druggable target [87].

While these recently-published comprehensive proteomics studies provide high-quality analytical data to classify breast cancers, they require fresh frozen tissue not typically available from standard surgical pathology procedures, limitations that hinder translation of their approach into the clinical setting. When compared to fresh frozen tissue, formalin-fixed paraffin-embedded (FFPE) specimens are widely-available and represent the format routinely used for patient diagnosis. Thus, they have the advantage of a long preservation in tumor repositories allowing for a direct linkage to clinical outcomes, making them a valuable resource for the development of clinically-applicable classifiers. In this regard, a 2022 study performed a comprehensive proteomic profiling of 75 FFPE breast cancer surgical specimens representing each of the four major PAM50 subtypes, from a cohort of patients receiving standard-of-care treatments and linked to clinical outcomes [60]. Specifically, the strategy utilized a protocol termed Single-Pot, Solid-Phase-enhanced, Sample Preparation Clinical Tissue Proteomics (SP3-CTP) [88–90] that has several strengths: it uses paramagnetic beads during protein digestion and clean-up, is performed in a single tube which results in minimal sample loss [90, 91], and has proven flexibility using different detergents, chaotropes, salts and isobaric tags that makes it applicable to a lower input quantity of FFPE specimens [89]. This strategy was further combined with isoDoping methodology that facilitates detection of low-abundance proteins that could be missed when using discovery-based data-dependent acquisition mode mass spectrometry approaches that select the most abundant peptide peaks [60]. Using 11-multiplex tandem mass tags that facilitate accurate quantification of proteins, this approach demonstrated the existence of four distinct proteome subgroups within 88 FFPE TNBC clinical specimens [60]. These four main proteomic subgroups displayed protein expression profiles, biological features and clinical outcomes that are consistent with the four best established RNA-based TNBC subtypes: BLIA, BLIS, LAR, and mesenchymal [7]. The TNBC proteomic subgroups and their biological features were further validated when applying the top 25% most highly-variant proteins from this classifier onto the 28 TNBC cases included in the CPTAC breast cancer study [85].

Transcriptomic and proteomic classifiers of triple negative breast cancer are summarized in Table 1.

**Table 1:** Transcriptomic and proteomic classifiers of triple negative breast cancer

**TNBC** **Classifier**	**Year**	**Number of TNBC cases**	**Subtypes**	**Biological level information**	**Key clinical findings**
**Transcriptomic profiling**					
Lehmann et al.[4] Lehmann et al. [57]	2011 Refined in 2016	587	**6 main subtypes:** Basal-like 1 Basal-like 2 Immunomodulatory Luminal androgen receptor Mesenchymal Mesenchymal stem-like	RNA expression	Basal-like 1 had a higher pathologic complete response rate after neoadjuvant chemotherapy and a better overall survival
			**4 main subtypes:** Basal-like 1 Basal-like 2 Luminal androgen receptor Mesenchymal		
Burstein et al. [7]	2015	198	**4 main subtypes:** Basal-like immune activated Basal-like immune suppressed Luminal androgen receptor Mesenchymal	RNA expression and DNA copy number	Four clinically distinct subtypes Survival outcomes were most favorable for basal-like immune activated and worst for basal-like immune suppressed
Jiang et al.[6]	2019	504	**4 main subtypes:** Immunomodulatory Basal-like immune suppressed Luminal androgen receptor Mesenchymal	RNA expression, DNA copy number and somatic mutations	Distinct patterns related to the Chinese TNBC population: higher frequencies of *PIK3CA* mutations and luminal androgen receptor subtype Basal-like immune suppressed with high-homologous recombination deficiency scores had a better prognosis when compared to those with low scores
Bareche et al. [35]	2018	550	**5 main subtypes:** Basal-like 1 Immunomodulatory Luminal androgen receptor Mesenchymal Mesenchymal stem-like	RNA expression, DNA copy number and somatic mutations	Immunomodulatory subtype was significantly associated with a better prognosis Luminal androgen receptor and mesenchymal stem-like subtypes were associated with low grade tumors
**Proteomic profiling**					
Gong et al. [76]	2022	90 fresh- frozen	**4 main subtypes** Immunomodulatory Basal-like immune suppressed Luminal androgen receptor Mesenchymal	Global proteomics and phospho-proteomics RNA expression, DNA copy number and somatic mutations	Four clinically distinct subtypes The proteome subtype that resembled immunomodulatory had the best survival while the proteome subtype that resembled luminal androgen receptor had the worst survival Potential therapeutic targets involved in fatty acid metabolism (e.g. FASN) specifically for the proteome subtype that resembled luminal androgen receptor A potential therapeutic target of NAE1 for the proteome subtype that resembled basal-like immune suppressed
Asleh et al. [60]	2022	88 Formalin- fixed paraffin- embedded	**4 main subtypes:** Basal-like immune activated Basal-like immune suppressed Luminal androgen receptor Mesenchymal	Global proteomics	Four clinically distinct subtypes Survival outcomes were most favorable for the proteome subtype that resembled basal-like immune activated and worst for basal-like immune suppressed Potential therapeutic targets involved in antigen presentation (e.g., TAP1, HLA-DQA1) for the proteome subtype that resembled basal-like immune activated Potential therapeutic targets involved in fatty acid metabolism (e.g., FASN) for the proteome subtype that resembled luminal androgen receptor

*Abbreviations*: *TNBC* triple negative breast cancer

While the recent TNBC proteomic classifiers have largely recapitulated previous observations at the transcriptomic level, they can more directly guide the development of protein-based clinical tests to distinguish TNBC subtypes and match patients to therapies. Proteomic techniques with the ability to map post-translational modifications are essential to identify therapeutic targets for TNBC subtypes and highlight the limitations of making inferences from RNA expression data. Modifications at the post-translational levels, such as phosphorylation, acetylation, and ubiquitination are known to impact the quantitative readout of protein levels resulting in a poor mRNA-protein correlation [92].

The integration of phospho-proteomics data in TNBC subtyping is critical as it identifies unique subtype-specific targetable pathways that often involve kinase cascades which are activated or deactivated by the reversible addition and removal of phosphate groups, with many of these kinases serving as drug targets [92]. The recent attempts by Lehmann et al.[58] and Gong et al.[76] had characterized protein signalling pathways that drive each TNBC transcriptomic and proteomic subtype by measuring phosphorylation of key residues in proteins pathways including DNA repair/cell cycle, PI3K/AKT, MAPK, antigen presentation and immune signalling highlighting the potential clinical value of integrating phospho-proteomics in current genomic and proteomic TNBC classifiers. In particular, the mesenchymal TNBC subtype was characterized with DNA repair signalling, as demonstrated by elevated phosphorylation of ATR and ATM which was not observed at the RNA level [58]. The LAR subtype was characterized by increased activity of PI3K/AKT pathway as demonstrated by high phosphorylation of AKT1 and AKT2 [58], high phosphorylation of the RB protein while retaining low levels of E2F [58], and an elevated phosphorylation of the MAP2K4 kinase which was notably observed as downregulated in LAR at the RNA level [76]. An increased activity of AR, its target SREBF1 and proteins involved in fatty acid metabolism, as inferred from phospho-proteomic data, was further characteristic of the LAR subtype [76]. BLIS tumors displayed high phosphorylation of CDK1 and VRK1 [76], suggesting that they may benefit from CDK1/2 inhibitors. BLIA tumors were characterized with a high expression of the kinases PKN1 and PRKD2 and an increased activity of STAT family proteins (STAT1, STAT2, STAT5A) as inferred from transcriptional factor analysis of phospho-proteomic data [76]. Key phospho-proteomic characteristics of each TNBC are summarized in Table 2.

**Table 2:** Key protein signalling pathways and phosphorylated proteins characteristics of each triple negative breast cancer subtype as derived from phospho-proteomics

TNBC subtype	Key phospho-proteomic characteristics
Luminal androgen receptor	PI3K/AKT signalling (AKT1, AKT2) RB (retaining low levels of E2F) MAP2K4 Androgen receptor activity (AR, SREBF1) Fatty acid metabolism
Mesenchymal	DNA repair signalling **(**ATM, ATR)
Basal-like immune suppressed	Cell cycle (CDK1) VRK1
Basal-like immune activated	PKN1 PRKD2 STAT signalling (STAT1, STAT2, STAT5A)

Data in the table are aggregated from Lehmann et al. [58] and Gong et al. [76]. *Abbreviations*: *TNBC* triple negative breast cancer

Altogether, the integration of phospho-proteomics with transcriptomic and proteomic data could help identify targetable signalling pathways and guide the discovery of therapeutic targets in a unique subtype-specific manner for TNBC.

### Role of single cell sequencing in triple negative breast cancer subtyping

Breast cancer subtypes as currently derived from bulk omic analyses do not account for the complexity of tumor microenvironments containing carcinoma cells, immune subsets, and stroma compartments which could potentially influence predictive value. Single cell analysis unmasks critical information at the individual cell level that could be related to tumor ecosystems representing a promising way to allow a better characterization of the heterogeneity of TNBC and guide more effective clinical management.

#### Single cell RNA profiling

In 2017, Chung et al. performed single-cell RNA-sequencing (scRNA-seq) on 515 cells from 11 breast cancer patients and found that carcinoma cells displayed intratumoral heterogeneity regarding breast cancer subtypes and signalling pathways, while non-cancer cells were mostly immune cells that displayed 3 different clusters of T lymphocytes, B lymphocytes and macrophages [93]. Carcinoma cells from TNBC showed low cell-to-cell intratumoral correlation suggesting a higher degree of intratumoral heterogeneity when compared to other subtypes. Extensive intratumoral heterogeneity was further shown when individual TNBC carcinoma cells from a given patient were assigned to one or more of the different Lehmann TNBC subtypes. Moreover, the analysis of T lymphocytes in this study showed that TNBC tumors exhibited a regulatory, exhausted or a mix of cytotoxic and exhausted phenotypes which could have implications for immunotherapy response. Specifically, some T cells were characterized by the expression of *TIGIT* and *LAG3* rather than *PD1* suggesting that targeting these exhaustion biomarkers could be a better strategy than anti-PD1/PDL1 drugs. Another scRNA-seq study on > 1500 cells from six untreated primary TNBC tumors has further defined a subpopulation of TNBC cells with a gene signature related to glycosphingolipid and innate immunity pathways associated with poor outcomes [94].

A comprehensive single cell atlas of the diverse immune phenotypes in the breast tumor microenvironment has been proposed by Azizi et al., who performed scRNA-seq on 45,000 sorted immune cells from 8 breast tumors with matched normal breast tissue, blood, and lymph nodes [95]. Key findings identified 38 distinct T cell, 27 myeloid cell, 9 B cell and 9 NK cell clusters with an increased phenotypic volume for immune cells in the tumor microenvironment when compared to normal breast tissue. This phenotypic heterogeneity was characterized by a continuum of T cell states and the expression of genes reflecting a diverse local microenvironment within the tumor related to inflammation, hypoxia, nutrient supply, and expression of ligands for activating and inhibitory receptors.

Attempts to characterize T cell heterogeneity and identify T cell subpopulations associated with a better response to immunotherapy in TNBC have been performed in a scRNA-seq study by Savas and colleagues [96]. Key findings identified a CD8 + CD103 + tissue-resident memory cell subpopulation expressing high levels of immune checkpoint and effector genes that is associated with an improved survival in both breast cancer and melanoma [96]. Tumors with high tissue resident memory CD8 + T cells, but low terminally exhausted CD8 + T cells, have been associated with better survival across several cancer types in a pan-cancer scRNA-seq analysis [97]. Subpopulations of CD8-CXCL13 and CD4-CXCL13 T cells predicted atezolizumab benefit in a scRNA-seq study on 22 advanced TNBC patients [98]. Furthermore, microenvironments containing various abundances of specific myoepithelial, T cells, and epithelial cell types can confer differential treatment benefits for anthracyclines and taxanes in a scRNA-seq study of breast cancer [99].

Single cell signatures in a detailed transcriptional atlas of 130,246 cells from 26 breast tumors including 11 TNBCs have been recently reported to stratify breast cancers into 9 “ecotype” clusters [100]. These ecotypes are characterized by unique patterns of stromal and immune cell composition, distinct clinical outcomes and are partially associated with intrinsic subtypes.

Analysis by scRNA-seq methods has further illustrated the stromal heterogeneity of breast cancer which could be linked to immunotherapy resistance [101, 102]. Wu and colleagues performed scRNA-seq of 5 TNBCs reporting two cancer associated fibroblast (CAF) and two perivascular-like cell populations [101]. CAFs clustered into myofibroblast-like CAFs or inflammatory-like CAFs while perivascular-like clustered into differentiated or immature cells. Key findings showed that these different stromal subpopulations displayed distinct morphologies, spatial localization and functional properties regulating the extracellular matrix. Of note, the expression of gene signatures characteristic of inflammatory-like CAFs and differentiated perivascular-like cells were associated with cytotoxic T cell dysfunction and exclusion, respectively. These findings underscore the importance of the stromal-immune interactions as revealed by single cell analysis for the design of better strategies that could potentially overcome immunotherapy resistance in TNBC. Another scRNA-seq study conducted to characterize a subset of CAFs known as CAF-S1 (which mediates immunosuppression in breast cancer) revealed 8 different CAF-S1 clusters [102]. Among these, two clusters characterized by high expression of genes involved in extracellular matrix and TGFβ signalling were associated with an immunosuppressive environment and resistance to immunotherapy.

While scRNA-seq allows the untargeted simultaneous detection of hundreds of thousands of transcripts, it does not characterize the specific phenotypes of cells that could be directly linked to treatment response at the single cell level.

#### Single cell protein profiling

Current mass spectrometry methods do not often allow single cell analysis, and while flow cytometry assays have been widely used to evaluate protein expression at single cell resolution [103], they are limited by the number of epitopes that can be simultaneously assessed due to overlapping spectra of fluorophores [103]. The advent of mass cytometry techniques that enable labeling antibodies with metal isotopes has expanded the number of markers that can be analyzed simultaneously [104].

Using mass cytometry, Wagner et al. generated a comprehensive single cell atlas of breast cancer assessing a 35-marker immune cell-centric panel and 38-marker tumor cell-centric panel in 26 million cells from 144 breast tumor and 50 non-tumor tissues [105]. Findings of this study showed that all breast cancer subtypes displayed an individuality in cellular phenotypic composition where despite the co-existence of multiple tumor cell phenotypes, one phenotype was often dominant, supporting that sub-clonal fitness could underlie the differential treatment responses in breast cancer subtypes [105]. In the immune cell compartment, high levels of PDL1 + tumor associated macrophages and exhausted T cells were observed in high-grade estrogen receptor positive and negative tumors, suggesting that they might benefit from immunotherapies in breast cancer.

Recent approaches have been further used to map the spatial organization of different single cell phenotypes in breast cancer using imaging mass cytometry (IMC) [106, 107]. Jackson et al. used an IMC panel of 35 antibodies specific to breast histology to image samples from 281 tumors that represent all breast cancer subtypes [106]. Images were segmented into single cells, tumor, and stromal regions and authors defined 14 tumor cell metaclusters by hierarchical clustering of the tumor single-cell phenotypes. TNBC cases were distributed across six metaclusters. When mapping the cellular spatial organization of tumors, they were mostly dominated by a single tumor cell metacluster and a few community types, while some tumors consisting of multiple epithelial cellular metaclusters were localized to spatially distinct communities. These tumors displayed a higher spatiophenotypic heterogeneity and patients were characterized with poorer outcomes. This study further showed the presence of stromal cells in every clinical subtype to be at similar densities and tumor-stromal microenvironment communities were more informative for patient outcome than tumor or stromal phenotype contents alone.

IMC analyses of 483 samples from the METABRIC series using a panel of 37 antibodies identified cell phenotypes that fell broadly into the categories of epithelial, stromal, and immune cells [107]. The genomic subtypes of breast cancer in this study were characterized by diverse tumor ecosystems where basal-like tumors, which consisted mostly of TNBC, showed 3 different phenotypes with one enriched for hormone receptor negative cells and high Ki67; another phenotype enriched for epithelial cells expressing basal cytokeratins; and a phenotype associated with hypoxia which had *CD274* copy number gain and *B2M* loss that could be linked to immune resistance [107]. In addition, distinct patterns for stromal cell enrichment were observed in different breast cancer subtypes, and these were associated with distinct clinical outcomes. Basal-like subtype showed an enrichment for two myofibroblast cell phenotypes, T cell and macrophages, and had a higher abundance of homotypic relationship among both epithelial and stromal cells indicating a higher separation of these two compartments in basal-like when compared to other subtypes.

A recent study using 693 breast tumors from the METABRIC series further mapped tumor microenvironment structures in situ using IMC and multitiered spatial analysis and revealed 10 recurrent tumor microenvironment structures with distinct enrichment patterns among breast cancer subtypes [108]. While the epithelial cell diversity of basal-like tumors was found to be akin to other subtypes, it displayed a higher tumor microenvironment diversity. This study further showed that a tumor microenvironment suppressed expansion structure, enriched for Treg cells, cells expressing immune checkpoint proteins, and proliferating cells was mostly found in estrogen receptor negative tumors and could be linked to immunotherapy resistance.

Newer technologies such as multiplexed ion beam imaging by time-of-flight (MIBI-TOF) have been specifically used to depict the cellular composition and spatial arrangement of the tumor-immune microenvironment in TNBC. Keren et al., used MIBI-TOF to simultaneously quantify in situ expression of 36 proteins, including tumor and immune antigens and immunoregulatory proteins at sub-cellular resolution in 41 FFPE specimens from TNBC patients [109]. Key findings showed large differences between patients in both composition and total number of immune cells. TNBC cases with similarly sized immune infiltrates were divided into 3 different subtypes based on their spatial organization: cold (no infiltrate), mixed (immune cells mixed with tumor cells), and compartmentalized (immune cells spatially separated from tumor cells). A compartmentalized immune structure with PDL1 and IDO along the tumor-immune border was associated with better patient survival. MIBI-TOF has been further used to depict changes in the tumor microenvironment that are associated with invasive relapse in ductal carcinoma in situ which is influenced by myoepithelial morphology [110].

Altogether, these studies emphasize the importance of integrating the tumor microenvironment in current TNBC subtyping to uncover relational features of tumor, stromal and immune cells that could be associated with distinct clinical outcomes”.

### Treatment of TNBC in the context of subtype knowledge: Recent developments from major clinical trials

The subtypes that underpin TNBC have not yet been prospectively incorporated into clinical trial designs, in part because breaking down TNBC yields smaller numbers of patients for each subtype delaying patient accrual. These issues intersect with endorsing molecular TNBC subtypes in clinical practice guidelines requiring more evidence to still be gathered to refine the definition of TNBC subsets most likely to benefit from specific therapies. Currently, the great majority of TNBC patients are treated with cytotoxic chemotherapy, mostly containing anthracyclines and taxanes [3]‒ based on evidence drawn from clinical trials including unselected TNBC populations [9].

Since defective DNA damage repair mechanisms are a hallmark of TNBC [32, 111, 112], the activity of DNA damaging agents, particularly platinum-based chemotherapy, has been investigated. Clinical and preclinical evidence have shown that the capacity of platinum salts to induce DNA crosslinks and strand breaks is enhanced in TNBCs inherently defective for DNA repair mechanisms such as HRD [113, 114] including those with *BRCA1/2* mutations [56, 115, 116].

In clinical practice, the activity of platinum-based drugs in TNBC has been demonstrated in the neoadjuvant setting [46, 117–119]. Two main trials (GeparSixto and CALGB40603) that randomized TNBC patients to receive standard neoadjuvant chemotherapy with or without carboplatin demonstrated a significant increase in pCR on the arm including carboplatin [117, 118]. Furthermore, results from the BrighTNess trial [46] comparing the addition of PARP inhibitors plus carboplatin or carboplatin alone to standard chemotherapy vs. an arm including standard chemotherapy only demonstrated that the two carboplatin-containing arms were the ones yielding higher pCR [46]. While these data support the inclusion of platinum-based drugs in the neoadjuvant setting for high-risk TNBC patients [46], associated toxicities suggest that their addition should be considered for a defined TNBC subset [120]. Attempts to test whether an increased pCR from neoadjuvant platinum-based drugs can be observed among TNBC patients with *BRCA1/2* germline mutations or other forms of HRD have reported conflicting results [71, 121–124]. These findings might be explained by the fact that tumors defective for DNA repair are still heterogeneous consisting of different TNBC subtypes including BLIA, BLIS, and some mesenchymal cases [125]. Thus, integration of DNA damage repair biomarkers along with other specific biomarkers may be a better approach to define the TNBC subtype benefitting most from platinum-based agents.

Achieving pCR has been shown to significantly improve the prognosis of TNBC patients [126]. For residual disease after neoadjuvant chemotherapy, the addition of adjuvant capecitabine resulted in longer disease-free survival and OS over control in the CREATE-X phase III clinical trial [127]. However, capecitabine brings additional toxicities and the TNBC subset that benefits from adjuvant capecitabine is yet to be determined.

In current practice, the standard therapy in metastatic breast cancer, including TNBC, is based on single-agent sequential chemotherapies rather than combination chemotherapies [3], as they have shown OS equivalent to combination chemotherapies, but with less toxicity and improved quality of life [128].

Liquid biopsy testing to match actionable mutations to targeted therapies is gaining momentum in the advanced setting [129, 130]. Plasma-based genotyping using a 73-gene panel (Guardant 360) has shown higher rates for identifying targetable mutations and subsequent matched therapies with improved survival compared to tissue-based genotyping, in metastatic breast cancer including TNBC [130]. Furthermore, serially monitored circulating tumor DNA-based detection of minimal residual disease burden after standard neoadjuvant therapies in high-risk early-stage TNBC could identify patients who benefit from further adjuvant therapies [131] (NCT04915755, NCT04849364, NCT04434040).

New targeted agents have emerged over the recent years in TNBC. While most trial designs did not prospectively incorporate TNBC subtypes, many of the molecular targets tested have biological mechanisms that support their activity in specific TNBC subtypes. In general, clinically-relevant molecular targets in TNBC can be categorized as targeting *BRCA*-mutated and BRCAness-associated tumors; targeting intracellular signalling pathways, particularly phosphoinositide 3-kinase (PI3K)/AKT and androgen signalling; targeting cell surface proteins; and targeting the immune microenvironment.

#### PARP inhibitors

PARP inhibitors have shown promising results in *BRCA*-mutated metastatic TNBC based on two major phase III trials, OlympiAD (olaparib) [132] and EMBRACA (talazoparib) [133], demonstrating a significant benefit for the primary endpoint of progression free survival (PFS) favoring PARP inhibitors over physician’s choice of chemotherapy in metastatic germline *BRCA*-mutated Her2 negative tumors (Table 3). Consistent with these results, another phase III trial, BROCADE 3, comparing veliparib vs. placebo administered with carboplatin and paclitaxel in germline *BRCA*-mutated Her2 negative tumors, has shown a PFS benefit favoring the veliparib arm [134] which further extended PFS as a maintenance monotherapy [135].

**Table 3:** Selected clinical trials assessing PARP inhibitors in triple negative breast cancer

Trial	Target	Treatment vs. control arm	Phase	Setting	Key results (Treatment vs. control)
**PARP inhibitors targeting germline *BRCA*-mutated tumors**					
OlympiAD [132]	PARP inhibition (olaparib)	Olaparib vs. Chemotherapy (physician’s choice)	III	Metastatic Her2-	PFS 7.0 vs. 4.2 months HR 0.58 (95%CI 0.43–0.80; *P* < 0.001)
EMBRACA [133]	PARP inhibition (talazoparib)	Talazoparib vs Chemotherapy (physician’s choice)	III	Metastatic Her2-	PFS 8.6 vs. 5.6 months HR 0.54 (95%CI 0.41–0.71; *P* < 0.001)
BROCADE 3 [134, 135]	PARP inhibition (veliparib)	Veliparib + chemotherapy vs Placebo + chemotherapy	III	Metastatic Her2-	PFS 14.5 months vs. 12.6 months HR 0.71 (95%CI 0.57–0.88; *P* = 0.002) For veliparib vs. placebo as a maintenance monotherapy PFS 25.7 months vs. 14.6 months HR 0.49 (95%CI 0.34–0.73; *P* < 0.001)
OlympiA [136]	PARP inhibition (olaparib)	Olaparib vs. Placebo	III	Adjuvant Her2-	3-year iDFS 85.9% vs. 77.1% HR 0.58 (95%CI 0.41–0.82; *P* < 0.001)
**PARP inhibitors targets beyond germline *BRCA*-mutated tumors**					
TBCRC048 [137]	PARP inhibition (olaparib)	Olaparib	II	Metastatic breast cancer Cohort (1): germline mutations in non-*BRCA1*/*2* homologous repair-related genes Cohort (2): somatic mutations in non-*BRCA1*/*2* homologous repair-related genes or somatic *BRCA1*/*2*	Objective response rate 82% among germline *PALB2* Objective response rate 50% among somatic *BRCA1/2* (primary endpoint met if objective response rate > 20%)
SWOG S1416 [138]	PARP inhibition (veliparib)	Veliparib + cisplatin vs. Placebo + cisplatin	II	Metastatic TNBC	PFS 5.7 months vs. 4.3 months HR 0.58, *P* = 0.02 among high HRD tumors

*Abbreviations*: *PFS* progression free survival, *iDFS* invasive disease-free survival, *HR* hazard ratio, *CI* confidence interval, *TNBC* triple negative breast cancer

The activity of PARP inhibitors has been recently demonstrated in the adjuvant setting showing that among germline *BRCA*-mutated Her2 negative tumors, olaparib was associated with a longer invasive disease-free survival when compared to placebo in the OlympiA trial [136].

The role of PARP inhibitors has been further assessed beyond *BRCA1/BRCA2* germline mutations. High response rates with PARP inhibitors have been observed among metastatic patients with germline *PALB2* mutations and somatic *BRCA1/BRCA2* mutations [137]. Moreover, high HRD scores have been associated with greater PFS from veliparib over placebo in metastatic TNBC, while results were not significant with germline *BRCA* tumors [138]. High *BRCA* genomic loss of heterozygosity scores were also associated with clinical benefit from rucaparib in the phase II RUBY trial [139]. However, these results are underpowered and require validation. Interestingly, recent analyses from both OlympiAD and EMBRACA according to HRD status did not reveal an association with PARP inhibitor benefit [140, 141], suggesting that other biomarkers beyond those captured by current genomic predictors may better characterize HRD. Ongoing trials including VIOLETTE (NCT03330847) and NOBROLA (NCT03367689) are testing PARP inhibitors using targeted sequencing panels of homology directed repair-related genes in non-germline *BRCA1/2* metastatic TNBC. Somatic mutations in such genes and high HRDetect scores have also been associated with higher response rates to neoadjuvant PARP inhibitors [53, 72]. RAD51 assessment in FFPE biopsies is an emerging biomarker for PARP inhibitor sensitivity and could be used in basal subtypes of TNBC [53, 72].

#### Targeting intracellular signalling pathways

Recent efforts have targeted intracellular signalling including PI3K/AKT and androgen pathways (Table 4). The PI3K/AKT pathway is often activated in TNBC and basal-like compared to luminal breast cancers despite having a much lower frequency of activating *PIK3CA* mutations (7% in basals vs. 43% in non-basal) [26]. Overactivity of PI3K/AKT is commonly achieved in TNBC through loss of negative regulators *PTEN* (35%) or *INPP4B* (30%) [26], suggesting AKT inhibitors are a more relevant approach than targeting *PIK3CA* in TNBC.

**Table 4:** Selected clinical trials targeting intracellular signalling pathways in triple negative breast cancer

Trial	Target	Treatment vs. control arm	Phase	Setting	Key results (Treatment vs. control)
**Strategies targeting the PI3K/AKT pathway**					
LOTUS [142]	AKT inhibitor (ipatasertib)	Ipatasertib + paclitaxel vs Placebo + paclitaxel	II	Advanced TNBC	ITT population PFS 6.2 months vs. 4.9 months HR 0.60 (95%CI 0.37–0.98; *P* = 0.037) *PI3K/AKT/PTEN* altered population PFS 9 months vs. 4.9 months HR 0.44 (95%CI 0.20–0.99; *P* = 0.04)
PAKT [143]	AKT inhibitor (capivasertib)	Capivasertib + paclitaxel vs Placebo + paclitaxel	II	Advanced TNBC	ITT population PFS 5.9 months vs. 4.2 months HR 0.74 (95%CI 0.50–1.08; *P* = 0.11) *PI3K/AKT/PTEN* altered population PFS 9.3 months vs. 3.7 months HR 0.30 (95%CI 0.11–0.79; *P* = 0.01)
IPATunity 130 [144]	AKT inhibitor (ipatasertib)	Ipatasertib + paclitaxel vs Placebo + paclitaxel	III	Advanced TNBC with *PI3K/AKT/PTEN* alterations	PFS 7.4 months vs. 6.1 months HR 1.02 (95%CI 0.71–1.45)
CAPItello 290 [145]	AKT inhibitor (capivasertib)	capivasertib + paclitaxel vs Placebo + paclitaxel	III	Advanced TNBC	Ongoing Primary endpoint is PFS Secondary endpoints include OS
**Strategies targeting androgen receptor signalling**					
TBCRC011 [147]	AR inhibitor (bicalutamide)	Bicalutamide	II	Advanced ER/PR negative breast cancer	6-month clinical benefit rate 19% (95%CI 7%-39%) Median PFS 1 month
MDV3100-11 [148]	AR inhibitor (enzalutamide)	Enzalutamide	II	Advanced TNBC	4-month clinical benefit rate 25% (95%CI 17%-33%) Median PFS 2.9 months
TBCRC032 [149]	AR inhibitor (enzalutamide) + PI3K inhibitor (taselisib)	Enzalutamide + taselisib vs enzalutamide alone	IB + II	Advanced TNBC with AR ≥ 10% by IHC	Evaluable patients on combination 4-month clinical benefit rate 35.7% while no clinical benefit on enzalutamide only Luminal androgen receptor TNBC subtype population 4-month clinical benefit rate (75% vs. 12.5%; *P* = 0.06) and (median PFS 4.6 months vs. 2 months; *P* = 0.08) compared to other TNBC subtypes

*Abbreviations*: *ITT* intention-to-treat, *PFS* progression free survival, *OS* overall survival, *HR* hazard ratio, *CI* confidence interval, *AR* androgen receptor, *IHC* immunohistochemistry, *TNBC* triple negative breast cancer

In the phase II LOTUS trial, a significant PFS benefit was observed with the AKT inhibitor ipatasertib in advanced TNBC [142] which was most pronounced in *PI3K/AKT/PTEN* altered tumors. These findings are consistent with another AKT inhibitor (capivasertib) in the phase II PAKT trial [143]. Considering the benefit observed in these two trials, the phase III IPATunity130 assessing ipatasertib among advanced TNBC with *PI3K/AKT/PTEN* alterations was designed (NCT03337724). However, results showed that IPATunity130 failed to validate the LOTUS observations [144]; biomarkers accounting for TNBC molecular subtypes could perhaps better define the subset benefitting from ipatasertib. CAPItello290 (NCT03997123), a phase III trial attempting to validate the PAKT observations, is ongoing [145]. In the neoadjuvant setting, phospho-AKT has been identified as a biomarker for improved pCR following paclitaxel with ipatasertib when compared to paclitaxel only in the FAIRLANE trial [146]. While results even extended to patients without *PIK3CA/AKT1/PTEN* alterations, they were not significant when adjusted for multiplicity (assessing 110 proteins) and hence require validation.

Strategies targeting androgen signalling have been also investigated, with two main agents: bicalutamide and enzalutamide. The TBCRC011 trial showed a 6-month clinical benefit rate of 19% among patients treated with bicalutamide with androgen receptor (AR) > 10% by IHC [147]. Enzalutamide has shown a 4-month clinical benefit rate of 25% among advanced TNBC patients with AR > 0% by IHC [148]. Combination therapy of androgen with PI3K inhibitors demonstrated a trend for a better clinical benefit rate among TNBC LAR tumors, while this response was not observed among those expressing AR in the TBCRC032 trial [149]. LAR subtype is genomically characterized by the expression of downstream targets other than AR itself [7] and to date there is no consensus on AR IHC assessment due to analytical variability issues [73, 150, 151]; thus, the best modality for defining AR positive tumors is yet to be identified. Overall, the correlative analysis of TBCRC032 demonstrates the value derived from a classifier that more reliably captures the biology and clinical behaviour of the LAR TNBC subtype, in comparison to a surrogate IHC biomarker based on AR assessment only.

#### Targeting cell surface proteins

Targeting cell surface proteins for selective delivery of potent agents is a treatment modality that has recently gained specific interest (Table **5**). The antibody drug conjugate targeting Trop-2, expressed on > 80% of TNBCs, is sacitizumab govitecan-hziy, a drug that conjugates an irinotecan metabolite payload to a monoclonal antibody with a cleavable linker [152]. Sacitizumab govitecan-hziy received accelerated approval for use in heavily pretreated metastatic TNBC patients in April 2020 [153]. Its significant clinical benefit was confirmed in the larger phase III ASCENT trial a year later [154]. Given that Trop-2 is expressed across the majority of TNBC cases (> 80%), it is less likely to map to any specific TNBC subtype. In support of this rationale, the *TACSTD2* gene (encoding TROP-2) was not found to be characteristic of any specific TNBC subtype as defined by different RNA and proteomic TNBC classifiers [4, 7, 60]. This suggests that targeting this protein may result in a similar effect in all TNBC patients.

**Table 5:** Selected clinical trials targeting cell surface proteins in triple negative breast cancer

Trial	Target	Treatment vs. control arm	Phase	Setting	Key results (Treatment vs. control)
**Strategies targeting cell surface receptors**					
IMMU-132–01 [153]	Trop-2 inhibitor (Sacitizumab govitecan-hziy)	Sacitizumab govitecan-hziy	I/II	Heavily pretreated metastatic TNBC	Objective response rate 33.3% Median PFS 5.5 months
ASCENT [154]	Trop-2 inhibitor (Sacitizumab govitecan-hziy)	Sacitizumab govitecan-hziy vs. Chemotherapy	III	Heavily pretreated metastatic TNBC	Median PFS 5.6 vs. 1.7 months HR 0.41 (95%CI 0.32–0.52; *P* < 0.001)
NCT01969643	LIV-1 Inhibitor (ladiratuzumab vedotin)	Ladiratuzumab vedotin	I	Advanced breast cancer	Ongoing Interim results: objective response rate of 32%
NCT03310957	LIV-1 Inhibitor (ladiratuzumab vedotin) + PD1 inhibitor (pembrolizumab)	Ladiratuzumab vedotin + pembrolizumab	I/II	Advanced TNBC	Ongoing Primary endpoint is objective response rate Secondary endpoints include PFS and OS
DESTINY-Breast04 [162]	Trastuzumab-deruxtecan (T-Dxd)	Trastuzumab-deruxtecan vs Chemotherapy (physician’s choice	III	Pretreated Her2-low, unresectable and/or metastatic breast cancer	Median PFS 9.9 vs. 5.1 months HR 0.51 (95%CI 0.40–0.64; *P* < 0.001)

*Abbreviations*: *PFS* progression free survival, *OS* overall survival, *HR* hazard ratio, *CI* confidence interval, *TNBC* triple negative breast cancer

Another antibody drug conjugate being investigated in heavily pretreated metastatic TNBC is ladiratuzumab vedotin, which links monomethyl auristatin E to a monoclonal antibody targeting LIV-1 protein [155]. Interim results showed an objective response rate of 32% [156] and this antibody drug conjugate is being assessed in combination with pembrolizumab as a first line treatment for advanced TNBC (NCT03310957). Both Trop-2 and LIV-1 are expressed across the majority of TNBC, agnostic to any specific TNBC subtype [7].

The antibody–drug conjugate of trastuzumab deruxtecan against Her2 has shown activity in clinically Her2 negative breast cancers with low Her2 expression (i.e., IHC 1 + or 2 + with lack of *ERBB2* amplification) [157, 158]. The prevalence of Her2-low tumors has been reported to constitute up to 38% of TNBC as a group [159–161] with the majority being classified as PAM50 basal-like [159, 161]. When compared to Her2-negative tumors, Her2-low are enriched for *ERBB2* and luminal-related genes within the subgroup of hormone positive breast cancers [161]. However, Her2-low tumors are biologically akin to Her2-negative within the TNBC subgroup [159, 161], suggesting that they can be agnostic to a specific TNBC subtype. Recently, the DESTINY-Breast04 randomized phase III trial investigating trastuzumab deruxtecan in all-comers pretreated Her2-low metastatic breast cancer patients showed a significant survival benefit favoring trastuzumab deruxtecan over physician’s choice of chemotherapy [162]. More data still need to be gathered to decide whether to incorporate TNBC subtypes in future trials testing trastuzumab deruxtecan.

#### Targeting the immune microenvironment

The recognition that many TNBCs have immune infiltrates has led to several trials evaluating immunotherapy, with emerging data showing BLIA is the subtype benefitting most from checkpoint blockade [13, 14]. Though several widely-used validated immune biomarkers (e.g., PD1, PDL1 and tumor infiltrating lymphocytes) approximate this TNBC subset, this is still a work in progress [163]. Several immune-related pathways and their cross-talk contribute to the immunogenicity of these tumors [105, 164]. Thus, the enumeration of single biomarkers seems insufficient to characterize the immune distinctions within TNBC that underlie diverse patient responses.

Two immune checkpoint inhibitors (pembrolizumab and atezolizumab) are approved for advanced TNBC (Table 6). In 2019, atezolizumab was the first to receive approval, based on results from the IMpassion130 trial showing that atezolizumab and nab-paclitaxel were superior to nab-paclitaxel with placebo in the intention-to-treat population and specifically among PDL1-positive tumors (defined by an immune cell score > 1% using the VENTANA SP142 assay) with advanced TNBC who had not received prior systemic therapy [10, 165].

**Table 6:** Selected phase III clinical trials assessing immune checkpoint inhibitors in the metastatic, neoadjuvant and adjuvant settings of triple negative breast cancer

Trial	Target	Treatment vs. control arm	Phase	Setting	Key results (Treatment vs. control)
**Targeting the immune microenvironment – Metastatic setting**					
IMpassion130 [10, 165]	PDL1 inhibition (atezolizumab)	Atezolizumab + nab-paclitaxel vs Placebo + nab-paclitaxel	III	Previously untreated TNBC	ITT population PFS 7.2 vs. 5.5 months HR 0.80 (95% CI 0.69- 0.92; *P* = 0.002) PDL1-positive population PFS 7.5 vs. 5 months HR 0.62 (95%CI 0.49–0.78; *P* < 0.001)
KEYNOTE-355 [12]	PD1 inhibition (pembrolizumab)	Pembrolizumab + chemotherapy (investigator’s choice) vs Placebo + chemotherapy (investigator’s choice)	III	Previously untreated TNBC	Combined positive score ≥ 10 population PFS 9.7 vs 5.6 months HR 0.65 (95%CI 0.49–0.86; *P* = 0.001)
**Targeting the immune microenvironment – Neoadjuvant setting**					
KEYNOTE-522 [168, 169]	PD1 inhibition (pembrolizumab)	Chemotherapy + pembrolizumab surgery pembrolizumab vs Chemotherapy + placebo surgery placebo	III	TNBC	ITT population pCR 63% vs. 55.6% PDL1-positive population pCR 68.9% vs. 54.9% PDL1-negative population pCR 45.3% vs. 30.3% Lymph node negative 64.8% vs. 44.1% Lymph node negative 64.9% vs 58.6% Event free survival at 36 months 84.5% vs. 76.8% HR 0.63 (95%CI 0.48–0.82; *P* < 0.001)
IMpassion031 [171]	PDL1 inhibition (atezolizumab)	Chemotherapy + atezolizumab surgery atezolizumab vs Chemotherapy + placebo surgery monitoring	III	TNBC	ITT population pCR 58% vs. 41% PDL1-positive population pCR 69% vs. 49%
NeoTRIPaPDL1 [174]	PDL1 inhibition (atezolizumab)	Chemotherapy + atezolizumab surgery chemotherapy vs Chemotherapy surgery chemotherapy	III	TNBC	ITT population pCR 43.5% vs. 40.8% PDL1-positive population pCR 51.9% vs 48% Event free survival primary endpoint results—pending
**Targeting the immune microenvironment – Adjuvant setting**					
ALEXANDRA/ IMpassion030 (NCT03498716)	PDL1 inhibition (atezolizumab)	Chemotherapy + atezolizumab vs Chemotherapy alone	III	TNBC	Ongoing Primary endpoint is iDFS Secondary endpoints include iDFS by PDL1 status, lymph node status and OS
SWOG S1418 (NCT02954874)	PD1 inhibition (pembrolizumab)	Pembrolizumab vs Observation	III	TNBC with residual disease measuring at least 1 cm in the breast and/or lymph node	Ongoing Primary endpoint is iDFS Secondary endpoints include OS and DFS
A-BRAVE (NCT02926196)	PDL1 inhibition (avelumab)	Avelumab vs Observation	III	TNBC	Ongoing Stratum A: surgery of the primary tumor followed by adjuvant chemotherapy Stratum B: residual disease after surgery of the primary tumor Primary endpoint is DFS Secondary endpoints include OS

*Abbreviations*: *ITT* intention-to-treat, *PFS* progression free survival, *HR* hazard ratio, *CI* confidence interval, *pCR* pathologic complete response, *DFS* disease-free survival, *iDFS* invasive disease-free survival, *OS* overall survival, *TNBC* triple negative breast cancer

A follow-up phase III trial, IMpassion131, which investigated the addition of atezolizumab to paclitaxel, was reported as negative for several proposed reasons such as a greater dose of steroids used when compared to IMpassion130 as a prerequisite for paclitaxel, the delivery of the backbone chemotherapy of nab-paclitaxel vs. paclitaxel in the tumor microenvironment, or notably due to TNBC heterogeneity [166]. Within the unselected category of TNBC in IMpassion131, the BLIA subtype represents a composition of immune features that may not be addressed by simple PDL1 tests. Indeed, a correlative analysis of cases from IMpassion131 demonstrated that TNBC patients classified as BLIA by RNA benefitted significantly from atezolizumab [14]. These findings corroborated recent observations from the IMpassion130 exploratory analysis showing that the RNA-based BLIA subtype was more predictive for atezolizumab benefit than PDL1 expression [13]. The phase III IMpassion132 trial is currently evaluating atezolizumab with capecitabine or gemcitabine for metastatic TNBC [167], but is not prospectively stratifying for the BLIA molecular subtype. Pembrolizumab received its full approval in July 2021 based on results from KEYNOTE-355 demonstrating that the combination of pembrolizumab with investigator’s choice chemotherapy was better than placebo with chemotherapy among patients whose tumors expressed high PDL1 by a different IHC test (combined positive score ≥ 10 using the 22C3 pharmDx assay) in previously untreated, advanced TNBC [12]. Inconsistencies and limitations of PDL1 IHC assays [163] and the superior clinical benefit of immune checkpoint inhibitors in the BLIA subtype as demonstrated in the correlative analyses of two major immunotherapy trials [13, 14] underscore the value of incorporating molecular TNBC subtypes for improved patient selection.

Immunotherapy has further revolutionized the neoadjuvant treatment of early-stage TNBC **(**Table**6****)**. In July 2021, the FDA granted an accelerated approval to pembrolizumab for the treatment of high-risk TNBC patients in combination with chemotherapy and then continued after surgery as a single-agent, based on findings from the KEYNOTE-522 trial [168]. Improved pCR rates and event free survival reported on the arm including pembrolizumab were consistent across both PDL1-positive and negative subgroups as defined by the SP142 assay [168, 169].

The finding that pembrolizumab benefit was also observed among PDL1-negative tumors provides evidence that matching TNBC BLIA tumors with immune checkpoint blockade would require additional or alternative biomarkers than PDL1 to more reliably approximate this subtype and establish a consensus on its best classifier. Such biomarkers further inform the selection of TNBC patients who still achieve the greatest benefit from immunotherapy as an extended adjuvant therapy when compared to current additional options (capecitabine and PARP inhibitors) approved in the same setting [127, 136, 170].

Other promising results in the neoadjuvant setting were recently observed in the phase III IMpassion031 trial showing higher pCR among TNBC patients receiving atezolizumab plus chemotherapy when compared to placebo plus chemotherapy, with pCR benefit more pronounced among PDL1-positive tumors [171]. While other trials have evaluated immunotherapy in the neoadjuvant setting including I-SPY2 [172], GeparNeuvo [173] and NeoTRIPaPDL1 [174], they showed inconsistent results, with some attributable to PDL1 analytical issues [173] and others pending event free survival [174]. The ongoing GeparDuoze trial is evaluating the addition of atezolizumab in the neoadjuvant setting of TNBC (NCT03281954). The choice of optimal immune priming neoadjuvant chemotherapy, its duration and the biological mechanisms governing responses require further investigation [175].

Currently, ongoing trials such as ALEXANDRA/IMpassion030 (NCT03498716), SWOG S1418 (NCT02954874) and A-BRAVE (NCT02926196) are evaluating the efficacy of adjuvant immunotherapy in TNBC **(**Table6**)**.

Combinations of immunotherapy with PARP inhibitors have garnered special interest in advanced TNBC. The phase II TOPACIO trial reported a 47% objective response rate among germline *BRCA*-mutated tumors treated with pembrolizumab and niraparib [176]. Furthermore, the phase II MEDIOLA trial evaluating durvalumab and olaparib showed a disease control rate of 80% at 12 weeks among patients with germline *BRCA*-mutated tumors [177]. Immune modulation induced by PARP inhibitors could be also relevant in BLIS tumors, and they can enhance the activity of immune checkpoint blockade irrespective of *BRCA1/2* mutation status [178].

### Identification of subtype-specific pharmacologic vulnerabilities in TNBC: Additional strategies for future investigation

Studies including TNBC cell line and animal models can identify pharmacologic vulnerabilities that may inform therapeutic strategies for TNBC patients. Their integration with multi-omic data further allows in silico analysis of genetic and pharmacologic screens in a subtype-specific context as recently demonstrated by a comprehensive study by Lehmann and colleagues [58]. This study identified 37 TNBC cell line models which had consensus TNBC subtyping for the basal-like 1, basal-like 2, mesenchymal and LAR categories across 5 different datasets. These cell lines were previously screened for genetic dependency and pharmacologic drug sensitivity to 250 compounds. In addition, this study identified 17 TNBC patient-derived tumor xenograft (PDTX) explant models treated with 96 compounds [58].

The LAR subtype was found to display genetic dependencies on AR signalling, PI3K/AKT/mTOR pathway, *ERBB2, CCND1* and *CDK4* [58]. LAR was characterized with unique drug sensitivities to the AR inhibitor bicalutamide, other drugs that inhibit the PI3K/mTOR pathway (GSK690693, ZSTK474, omipalsib, OSI-027, and AZD6482) [58], and CDK4/6 inhibitors (palbocilcib) [74]. Similar observations were found in the LAR PDTX models and thus support investigating these agents in clinical trials targeting the LAR TNBC subtype [58].

The basal-like 1 subtype displayed genetic dependencies on cell cycle and DNA repair and exhibited unique drug sensitivities to cell cycle inhibitors (ZM447439 and PHA-793887) and drugs targeting DNA repair (KU-559333 and NU7441) [58]. Similarly, the basal-like 1 PDTX models showed sensitivity to inhibitors of cell cycle (vinblastine, MLN8237 and BI-2536) and DNA repair (BMN-673, gemcitabine, and camptothecin). In contrast, the basal-like 2 subtype exhibited some genetic dependencies on DNA repair (*RAD50* and *TERF1*) while displaying a unique dependency on developmental genes and the MAPK pathway. Basal-like 2 tumors displayed a unique sensitivity to DNA repair inhibitors (olaparib and CP466722) and MAPK inhibitors (trametinib, PD0325901, refametinib, and selumetinib). Basal-like 2 PDTX models were found to be sensitive to DNA alkylating agents (carboplatin, temozolomide, and cyclophosphamide) [58].

The mesenchymal subtype showed dependencies on adhesion/motility genes, growth factor genes, and retinoic acid receptor alpha [58]. These tumors were sensitive to kinase inhibitors (midostaurin, BX796, SL0101 and ponatinib) and a retinoic acid receptor agonist (tretinoin). While mesenchymal PDTX were resistant to most compounds tested, they showed some sensitivity to inhibitors targeting developmental pathways such as TGFβRI inhibitor (SB-505124). Mesenchymal cell lines were further found to be specifically sensitive to the EZH2 inhibitor tazemetostat through restoration of major histocompatibility complex-I expression. This strategy has been further shown to enhance taxane sensitivity in murine xenograft TNBC mesenchymal model and could potentially augment immunotherapy efficacy [58]. These findings could be of important clinical relevance as the TNBC mesenchymal subtype has to date lacked a particular strategy for subtype-specific therapy. Characteristic subtype-specific therapeutic vulnerabilities in TNBC are shown in Table 7.

**Table 7:** Characteristic subtype-specific therapeutic vulnerabilities in TNBC

TNBC subtype	Pathway	Genetic dependency	Pharmacologic dependency	Patient-derived tumor xenograft dependency
Luminal androgen receptor	AR signalling	*AR* *SPDEF* *FOXA1*	Bicalutamide (AR)	Bicalutamide (AR)
	PI3K/AKT signalling	*PIK3CA* *AKT1* *EIF4A2* *RPS6* *ERBB2*	GSK690693 (AKT) ZSTK474 (PI3K) Omipalsib (PI3K/mTOR) OSI-027 (mTOR) AZD6482 (PI3Kb)	GDC0941 (PI3K) NVP-BEZ235 (PI3K/mTORC1/2) AZD8055 (mTORC1/2) Everolimus (mTOR)
	Other	*CDK4* *CCND1*	Palbociclib (CDK4/6)	
Basal-like 1	Cell cycle	*POLR1D* *POLD4* *CDC25A* *CDCA7* *BIRC3* *WEE1*	ZM447439 (AURKA/B) PHA-793887 (CDK2/5/7)	Vinblastine (Microtubules) MLN8237 ((AURKA) BI-2536 (PLK1/2/3) Bortezomib (Proteasome)
	DNA repair	*ATR* *HMCES* *LIG3*	KU-559333 (ATM) NU7441 (DNAPK)	BMN-673 (PARP1) Gemcitabine (DNA replication) Camptothecin (TOP1)
Basal-like 2	DNA repair	*RAD50 TERF1*	Olaparib (PARP) CP466722 (ATM)	Carboplatin (DNA akyl) Temozolomide (DNA akyl) Cyclophosphamide (DNA akyl)
	Developmental pathways	*WNT3* *JAG1* *NODAL BMPR1A RSPO2* *SMAD9*		
	MAPK signalling	*MAP3K1* *MAP2K4*	Trametinib (MEK1/2) PD0325901 (MEK1/2) Refametinib (MEK1/2) Slumetinib (MEK1/2)	
Mesenchymal	Adhesion/motility	*RAC1* *ITGAV* *RSPO1* *CD44* *ITGB3*	SL0101 (RSK)	TGFβRI inhibitor (SB-505124) JNK Inhibitor VIII (JNK) EHT 1864 (Rac GTPases) BIRB 0796 (p38/JNK2)
	Growth factor	*FGFR1* *FRS2* *NGFR*	Ponatinib (RTK) Midostaurin (kinase) BX796 (kinase)	Axitinib (multi − RTK)
	Retinoic acid receptor alpha	*RARA*	Tretinoin (RARA agonist)	
	Global hypomethylation and increased PRC2 activity Hypermethylation of immune response and antigen presentation promoter regions		Tazemetostat (EZH2 inhibitor) CPI-1205 (EZH2 inhibitor) MAK-683 (EED inhibitor)	Tazemetostat (EZH2 inhibitor) CPI-1205 (EZH2 inhibitor) MAK-683 (EED inhibitor)

Data in the table are aggregated from Lehmann et al. [58] and Asghar et al. [74]. *Abbreviations*: *TNBC* triple negative breast cancer, *PRC2* polycomb repressive complex 2, *RTK* receptor tyrosine kinase

Altogether, the recent identification of therapeutic vulnerabilities through integrative analyses of multi-omic data can inform future clinical trial design to investigate the efficacy of potential drugs in a subtype-specific context of TNBC.

## Conclusions: Incorporating TNBC molecular subtype into clinical trials

Overall, only the correlative analysis of the TBCRC032 trial has incorporated the molecular TNBC subtype classification (LAR) in its prospective design. The recent post-hoc exploratory analyses of IMpassion130 and IMpassion131 showing that BLIA RNA TNBC subtype predicts immunotherapy benefit better than PDL1 IHC assessment highlights the real advantage of integrating more detailed and quantitative multigene subtyping, challenging the design and interpretation of clinical trials that ignore the existence of distinct molecular TNBC subtypes and their treatment implications in the context of the molecular targets being tested.

When compared to RNA-based methods, the recently proposed proteomic classifiers that characterize TNBC into four main subtypes (and highly correlate with established RNA classifications) provide an advantage for translation to clinically-relevant diagnostic and predictive tests for immunotherapy. Furthermore, protein candidates that are enriched in the TNBC BLIA subtype, as profiled by proteomic classifiers, could identify candidate biomarkers in IHC panel designs matching patients to immunotherapy. Currently, clinical proteomic platforms are not present in diagnostic labs, and TNBC proteomic classifiers still requiring more validation on independent clinical datasets. In contrast, IHC remains the most commonly-used method despite its limitations. Given that resistance to immunotherapy in TNBC can be explained by the contribution of several immune-related pathways and their cross-talk to form an effective anti-tumor immune response, the potential integration of proteomics-derived IHC biomarkers concurrently with PD1/PDL1 panels could be leveraged for better matching of TNBC patients to immune checkpoint blockade until TNBC proteomic classifiers can be adapted into clinical practice.

Molecular subtyping and genomic biomarkers have further informed selections for targeted therapies and demonstrated improved clinical benefit rates among refractory, metastatic TNBC patients in the FUTURE umbrella trial [179]. The FUTURE-C PLUS trial further demonstrated the efficacy of anti-angiogenic tyrosine kinase inhibitor, anti-PD1 and nab-paclitaxel triple therapy in the BLIA subtype defined by IHC for CD8 [180] based on the rationale that angiogenic inhibitors enhance immune activity among immune cold tumors [181]. These promising results await validation in the larger FUTURE-SUPER trial (NCT04395989).

The recent integration of phospho-proteomic data to identify targetable signalling pathways in a subtype-specific manner can better reflect the biological function underpinning each genomic, transcriptomic, and proteomic TNBC subtype at the cellular level [58]. Thus, TNBC multi-omics analysis provides future insights into the mechanisms underlying patient’s response or resistance to therapy.

The advent of single cell profiling at both the RNA and protein level further illustrates the heterogeneity in the tumor microenvironment that still exists within current TNBC subtypes.

The findings from recent single cell analyses in breast cancer underscore the critical information that can be obtained at the individual cell level in relation to tumor ecosystems to improve current classifications and treatment options for TNBC patients.

Genomic and proteomic investigations of TNBC open opportunities to apply scientific knowledge to several unresolved issues in breast cancer: 1) Establishment of a consensus on the most relevant TNBC classification, platforms and informatics that can be applied in clinical settings and to map drug vulnerabilities in pre-clinical models, 2) Development of more accurate candidate protein-based biomarkers to improve current clinical tests particularly for approximating the immunotherapy-sensitive BLIA molecular subtype, 3) Incorporating molecular subtypes into prospective clinical trial designs to establish the clinical utility of biomarkers distilled from modern ‘omics research, and 4) Investigating the utility of single cell profiling analysis of the tumor microenvironment to build an ecosystem-based patient classification that could refine current TNBC classifiers.

Ultimately, the translation of this knowledge into tests with clinical utility to improve patient care requires a systematic approach to generate a high level of evidence [8, 182–184]: building from discovery-based tests, optimizing analytically-valid methods, and testing prespecified hypotheses on at least two similar independent clinical trials to establish clinical validity. The correlative work done over the past year has partially completed this process for immunotherapy trials of TNBC, in what are still early days in our exciting and dynamic era of precision oncology.

## Data Availability

Not applicable.

## Abbreviations

APM: Antigen processing machinery
AR: Androgen receptor
BLIA: Basal immune activated
BLIS: Basal-like immune activated
CAF: Cancer associated fibroblast
CALGB: Cancer and Leukemia Group B
CI: Confidence interval
CIN: Chromosomal instability
CPTAC: Clinical Proteomic Tumor Analysis Consortium
DC: Dendritic cells
DFS: Disease-free survival
DNA: Deoxyribonucleic acid
EGFR: Epidermal growth factor receptor
ER: Estrogen receptor
FDA: Food and Drug Administration
FFPE: Formalin-fixed paraffin-embedded
Her2: Human epidermal growth factor receptor-2 protein
HR: Hazard ratio
HRD: Homologous recombination deficiency
iDFS: Invasive disease-free survival
IFN: Interferon
IHC: Immunohistochemistry
IMC: Imaging mass cytometry
ITT: Intention-to-treat
LAR: Luminal androgen receptor
METABRIC: Molecular Taxonomy of Breast Cancer International Consortium
MSL: Mesenchymal stem-like
MIBI-TOF: Multiplexed ion beam imaging by time-of-flight
NK: Natural killer
OS: Overall survival
PAM50: Prediction Analysis of Microarrays 50
PARP: Poly (ADP-ribose) polymerase
pCR: Pathologic complete response
PDL1: Programmed death ligand 1
PDTX: Patient-derived tumor xenograft
PFS: Progression free survival
PI3K: Phosphoinositide 3-kinase
PR: Progesterone receptor
RNA: Ribonucleic acid
SP3-CTP: Single-Pot, Solid-Phase-enhanced, Sample Preparation-Clinical Tissue Proteomics
TBCRC: Translational Breast Cancer Research Consortium
TCGA: The Cancer Genome Atlas
TILs: Tumor infiltrating lymphocytes
TNBC: Triple negative breast cancer
